# Regulation of angiotensin II type 1 receptor expression in ovarian cancer: a potential role for BRCA1

**DOI:** 10.1186/1757-2215-6-89

**Published:** 2013-12-09

**Authors:** Fang-Fang Bi, Da Li, Chen Cao, Chun-Yan Li, Qing Yang

**Affiliations:** 1 Department of Obstetrics and Gynecology, Shengjing Hospital, China Medical University, Shenyang 110004, China; 2 Department of Pathology, Chinese PLA General Hospital, Beijing 100853, China; 3 Department of Histology and Embryology, Institute of Basic Medical Sciences, Chinese Academy of Medical Sciences, School of Basic Medicine, Peking Union Medical College, Beijing 100005, China

**Keywords:** *BRCA1*, Angiotensin II type 1 receptor, Ovarian cancer

## Abstract

**Background:**

Both *BRCA1* and angiotensin II type 1 receptor (*AGTR1*) play a critical role in ovarian cancer progression. However, the crosstalk between *BRCA1* and *AGTR1* signaling pathways remains largely unknown.

**Methods:**

*BRCA1* promoter methylation was analyzed by bisulfite sequence using primers focused on the core promoter region. Expression levels of *BRCA1* and *AGTR1* were assessed by immunohistochemistry and real-time PCR. Regression analysis was used to examine the possible relationship between *BRCA1* and *AGTR1* protein levels. Knockdown or overexpression of *BRCA1* was achieved by using a lentiviral vector in 293 T cells and SKOV3 ovarian carcinoma cells, and primary non-mutated and *BRCA1*-mutated ovarian cancer cells.

**Results:**

*BRCA1* dysfunction (*BRCA1* mutation or hypermethylated *BRCA1* promoter) ovarian cancer showed decreased *AGTR1* levels compared to normal tissue. In contrast, *AGTR1* expression was increased in non-*BRCA1*-mutated ovarian cancer. Notably, *BRCA1* activation was an effective way to induce *AGTR1* expression in primary ovarian cancer cells and a positive correlation exists between *BRCA1* and *AGTR1* expression in human ovarian cancer specimens.

**Conclusions:**

These results indicate that *BRCA1* may be a potential trigger involved in the transcriptional regulation of *AGTR1* in the development of ovarian cancer.

## Background

Ovarian cancer is the most lethal gynecological malignancy in women worldwide [1]. To date, although the exact cause of ovarian cancer remains largely unknown, BRCA mutations are the main known hereditary factor [2], and the risk of ovarian cancer conferred by BRCA mutations can be regulated by both genetic and environmental components [3]. The angiotensin II type 1 receptor (*AGTR1*) is a novel component of the renin-angiotensin system, and has a direct effect on blood pressure and heart hypertrophy [4]. Recently, *AGTR1* has drawn considerable interest, not only in the field of cardiovascular risk but also in several types of gynecological malignancies, such as endometrial cancer [5,6], cervical carcinoma [7], and especially ovarian cancer [8-10]. Accumulating evidence also indicates that an increased risk of ovarian cancer and poor patient outcome are associated with *AGTR1* expression [9,11]. Our previous study has found that *AGTR1* interacts with genetic and environmental factors, which exert a potent effect on the proliferation and survival of the estrogen-induced Ishikawa cell line [12]. Several recent studies also support a possible role for *AGTR1* in regulating cell proliferation during cancer development [13]. Additionally, an increasing amount of evidence suggests that *BRCA1* haploinsufficiency mutations are more likely to result in cancer, due to an extraordinary ability for clonal growth and proliferation [14]. However, the complex interrelationship between *AGTR1* and *BRCA1* remains to be elucidated. Therefore, the present study was undertaken to investigate *AGTR1* expression from genetic (*BRCA1* mutated or not) and epigenetic (*BRCA1* promoter methylated or not) aspects in ovarian cancer, and to provide novel insights into the regulatory mechanism of *AGTR1*.

## Methods

### Patients and tissue collection

This study was approved by the Institutional Review Board at China Medical University. Serous ovarian cancer patients were enrolled between 2010 and 2012, and all patients gave informed consent. Fresh tumor samples, adjacent normal ovarian tissues, ascites and blood samples were obtained at the time of primary surgery before any chemotherapy or radiotherapy. Hematoxylin and eosin staining of the samples for histopathological diagnosis and grading were determined by three staff pathologists using the World Health Organization criteria. All patients were screened for *BRCA1* mutations by multiplex polymerase chain reaction (PCR) with complete sequence analysis using methods reported by Bi and Simard [15,16], Their characteristics are given in Additional file 1: Table S1.

### Cell culture and lentiviral transfection

Primary ovarian cancer cells were obtained from ascites for 15 *BRCA1*-mutated and 15 non-mutated patients undergoing surgery for ovarian cancer and cultured in RPMI 1640 with 10% fetal bovine serum (Invitrogen, CA USA) using methods reported by Szlosarek [17]. Primary ovarian cancer cells used in all experiments were passage 2. The proliferation rate is shown in Additional file 2: Figure S1 (methods shown in Additional file 3). Human 293 T cells and SKOV3 ovarian carcinoma cells were maintained in DMEM with 10% fetal bovine serum (Invitrogen). Each experiment was repeated four times for primary ovarian cancer cells of each patient, 293 T cells and SKOV3 cells. Lentiviral vectors expressing short hairpin RNAs (shRNAs) against *BRCA1* (NM_007299) were obtained from GeneChem Co., Ltd (Shanghai, China), and synthesized as follows: Forward: 5′-CCGGAACCTGTCTCCACAAAGTGTGCTCGAGCACACTTTGT GGAGACAGGTTTTTTTG-3′, and Reverse: 5′-AATTCAAAAAAACCTGTCTCCACAAAGTGTGCTCGAGCACACTTTGTGGAGACAGGTT-3′. The non-silencing siRNA sequence (TTCTCCGAACGTGTCACGT) was used as a negative control. For overexpression of *BRCA1*, the open reading frame of *BRCA1* (NM_007299) was cloned into the lentiviral vector GV287 (Ubi-MCS-3FLAG-SV40-EGFP) (GeneChem, Shanghai, China). Transfections were performed using polybrene and enhanced infection solution (GeneChem) according to the manufacturer’s recommended protocol. The efficiency of *BRCA1* knockdown and overexpression is shown in Additional file 4: Figure S2 (methods shown in Additional file 3).

### Real-time quantitative PCR

Total RNA was extracted using Trizol reagents (Invitrogen) according to the manufacturer’s protocol. DNA contamination was removed by adding DNase I (Invitrogen) according to the manufacturer’s protocols. Total RNA was then reverse-transcribed from 2 μg of RNA using the PrimeScript RT Master Mix kit (TaKaRa, Dalian, China) and amplified by SYBR Premix Ex TaqTM II (TaKaRa) in a Roche LightCycler 2.0 instrument (Roche Diagnostics, Mannheim, Germany). The specific primer sequences were as follows: *AGTR1*: 5′-CCTCAGATAATGTAAGCTCATCCAC-3′ (F) and 5′-GCTGCAGAGGAATGTTCTCTT-3′ (R); *BRCA1*: 5′-GGCTATCCTCTCAGAGTGACATTT-3′ (F) and 5′-GCTTTATCAGGTTATGTTGCATGG-3′ (R); GAPDH: 5′-AGGTGAAGGTCGGAGTCA-3′ (F) and 5′-GGTCATTGATGGCAACAA-3′(R).

GAPDH mRNA was amplified as an internal control for normalization of each sample. All samples were analyzed in triplicate using the 2^–∆∆CT^ method.

### Immunohistochemistry

The standard SP kit (Zhongshan, Beijing, China) was used for immunohistochemical staining. Briefly, serial 4-μm sections were obtained from each paraffin-embedded tissue block. Following deparaffinization and rehydration, sections were subjected to microwave antigen retrieval. The primary antibody were rabbit polyclonal anti-AGTR1 (sc-1173) (1:100; Santa, Cruz Biotechnologies, USA) and rabbit polyclonal anti-BRCA1 (sc-642) (1:100; Santa), and the sections were incubated overnight at 4°C with this antibody. 3,3‘-diaminobenzidine was used as the chromogen. Nuclei were counterstained with hematoxylin, and slides were dried and mounted. Negative controls were incubated with phosphate-buffered saline instead of the antibody. Immunostaining was evaluated by two independent pathologists, blinded to the identity of subject groups. Area quantification was made with a light microscope at a magnification of 400 × and analyzed by Image-Pro Plus 6.0 (Media 2 Cybernetics, USA). Intensity of the staining was divided into 10 units.

### Bisulfite sequencing for *BRCA1* promoter

All the tissues were used for bisulfite sequencing from the non-*BRCA1*-mutated cases. Genomic DNA extracted from ovarian cancer and normal ovarian tissue with a TIANamp Genomic DNA kit (Tiangen biotech, Beijing, China) was subjected to bisulfite conversion using the EZ DNA Methylation-Direct kit (Zymo research, Orange, USA) following the manufacturer’s instructions; the conversion efficiency was estimated to be at least 99.6%. It was then amplified by nested PCR. After gel purification, cloning and transformation into *E. coli* Competent Cells JM109 (TaKaRa), ten positive clones of each sample were sequenced to ascertain the methylation patterns of each CpG locus. The following primers were used for *BRCA1* gene (Accession number: NG_005905; GRCh37/hg19) promoter: round I, F: 5′-TTGTAGTTTTTTTAAAGAGT-3′ and R: 5′-TACTACCTTTACCCAAAACAAAA-3′; and round II, F: 5′-GTAGTTTTTTTAAAGAGTTGTA-3′ and R: 5′-ACCTTTACCCAAAACAAAAA-3′. The conditions were as follows: 95°C for 2 min, 40 cycles of 30s at 95°C, 30s at 56°C and 45 s at 72°C, then 72°C for 7 min.

### Statistical analysis

Regression analysis was used to examine the possible relationship between *AGTR1* and *BRCA1* expression. The data are presented as means ± SD. Statistical differences in the data were evaluated by Student’s *t* test or one-way ANOVA as appropriate, and were considered significant at *P* < 0.05.

## Results

### *BRCA1* can regulate *AGTR1* expression in primary ovarian cancer cells

**Figure 1:**
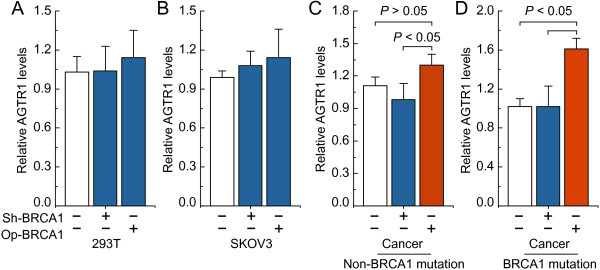
**Effects of *BRCA1 *on *AGTR1 *expression. A**-**D**, relative AGTR1 mRNA levels after overexpression or knockdown of BRCA1 in 293 T cells, human SKOV3 ovarian carcinoma cells, and primary non-mutated and BRCA1-mutated ovarian cancer cells. Bar graphs show mean ± SD. Sh, shRNAs; Op, overexpression.

To confirm the role of *BRCA1* in the regulation of *AGTR1*, the effects of overexpression or knockdown of *BRCA1* were observed in 293 T cells, human ovarian carcinoma cell line SKOV3, primary ovarian cancer cells with identified *BRCA1* mutations or non-mutation. The results indicated that there were no significant changes in the expression of *AGTR1* after overexpression or knockdown of *BRCA1* in 293 T and SKOV3 cells (Figure 1A-B). Interestingly, we observed that overexpression of *BRCA1* was an effective way to induce an increase in *AGTR1* levels in primary non-mutated and *BRCA1*-mutated ovarian cancer cells, although *AGTR1* levels were not sensitive to the *BRCA1* knockdown (Figure 1C-D).

### Differences in expression patterns of *AGTR1* in *BRCA1*-mutated and non-mutated ovarian cancer

**Figure 2:**
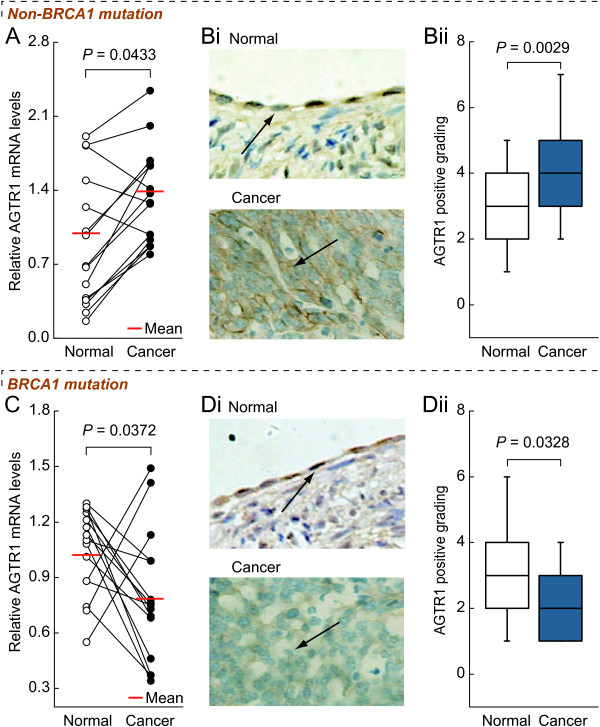
***AGTR1 *expression patterns in *BRCA1*-mutated and non-mutated ovarian cancer*. *A** and **C**, relative AGTR1 mRNA levels were measured in non-mutated or BRCA1-mutated ovarian cancer compared to their adjacent normal tissue, respectively (each group, n = 15). Bi and Di, sections were subjected to immunostaining for AGTR1 in non-mutated or BRCA1-mutated ovarian cancer compared to their adjacent normal tissue, respectively (each group, n = 15). Arrow shows positive staining for AGTR1 in the membrane. Bii and Dii, summary of the percentages of AGTR1-positive cells from the measurements shown in Bi and Di, respectively. Intensity of the staining was divided into 10 units. Magnification is 400 ×.

Real-time PCR and immunohistochemical analysis showed that the levels of *AGTR1* mRNA and protein were increased in non-*BRCA1*-mutated ovarian cancer compared to adjacent normal tissue (Figure 2A and B, *P* < 0.05). It is, however, interesting to note that *BRCA1*-mutated ovarian cancer as compared to their adjacent normal tissue reduced the expression of *AGTR1* (Figure 2C and D, *P* < 0.05).

### Hypermethylated *BRCA1* promoter-mediated decreased expression of *BRCA1* is correlated with *AGTR1* levels

**Figure 3:**
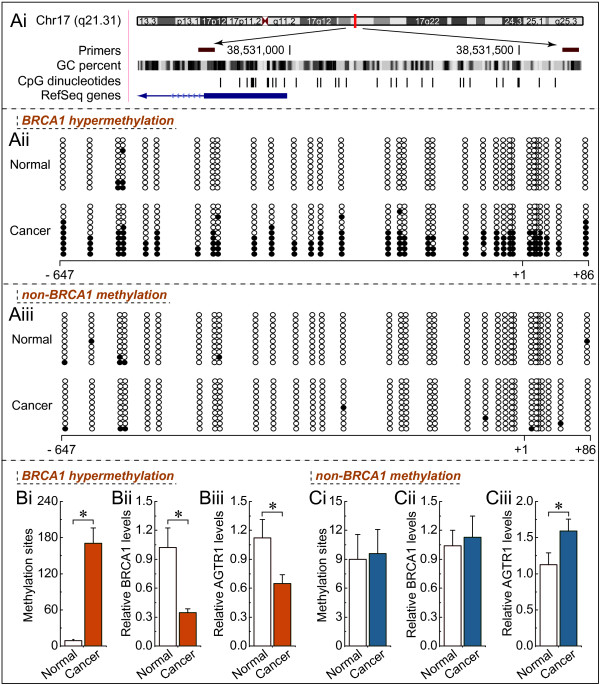
***AGTR1 *expression patterns in ovarian cancer with hypermethylated promoter-mediated *BRCA1 *dysfunction*.*** Ai, location of CpG sites in the core promoter region of BRCA1. Genomic coordinates are shown, along with the primer-amplified fragments, GC percentage, location of individual CpG dinucleotides (dashes) and BRCA1 RefSeq gene (exon 1 shown as a blue box and intron shown as an arrowed line). The arrow indicates the direction of transcription. Aii and Aiii, comparative analysis of methylation patterns in the core promoter region of BRCA1 and their adjacent normal tissue (each group, n = 15). The circles correspond to the CpG sites denoted by black dashes in Figure 3Ai. Closed circles, methylation; open circles, unmethylated. Ten individual clones were sequenced for each sample. Bi and Ci, summary of the methylation levels of BRCA1 core promoter from the measurements shown in Aii and Aiii, respectively. Bii and Cii, relative BRCA1 mRNA levels were measured in ovarian cancer with identified hypermethylated or unmethylated BRCA1 promoter compared to their adjacent normal tissue, respectively (each group, n = 15). Biii and Ciii, relative AGTR1 mRNA levels were measured in ovarian cancer in the presence or absence of BRCA1 dysfunction, respectively (each group, n = 15). Bar graphs show mean ± SD. *P < 0.05 vs. Normal.

In mammals, promoter methylation at CpG dinucleotides is an important feature regulating gene expression [18]. Consistent with this idea, we showed that ovarian cancer tissue with a hypermethylated *BRCA1* promoter displayed decreased expression of *BRCA1* in comparison with adjacent normal tissue (Figure 3Aii and Bi*, P* < 0.05). However, no significant *BRCA1* expression differences were observed in ovarian cancer with unmethylated *BRCA1* promoter as compared to adjacent normal tissue (Figure 3Aiii and Ci*, P* > 0.05). Based on these considerations, the low levels of *BRCA1* appeared to be mediated by promoter hypermethylation, making this an appropriate model to investigate the physiological relationship between *BRCA1* and *AGTR1*. Notably, the expression levels of *AGTR1* were decreased markedly (Figure 3Biii), along with hypermethylated promoter-mediated *BRCA1* deficiency in ovarian cancer (Figure 3Bii). In contrast, *AGTR1* expression was increased in ovarian cancer tissue (Figure 3Ciii), along with no significant difference of *BRCA1* promoter methylation and expression (Figure 3Ci and Cii).

### *AGTR1* is positively correlated with *BRCA1* expression in ovarian cancer samples

**Figure 4:**
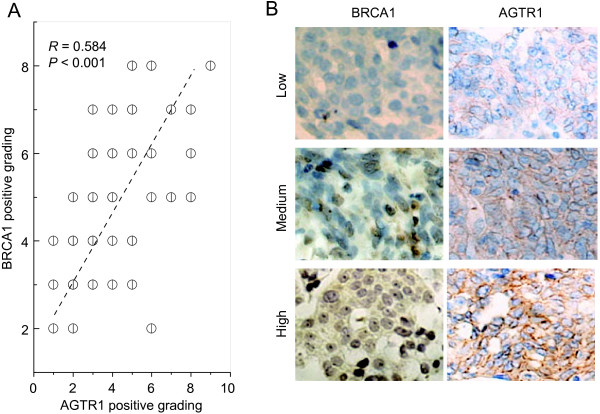
**Correlation between the expression levels of *BRCA1 *and *AGTR1 *in ovarian cancer samples. A**, Correlation between the BRCA1 and AGTR1 protein levels in 63 human ovarian cancer tissue samples. Intensity of staining was divided into 10 units. **B**, Examples of immunohistochemical staining showing the positive correlation between the expression levels of BRCA1 and AGTR1 in ovarian cancers. Magnification is 400 ×.

Of particular interest and potential clinical relevance, the relationship between *BRCA1* and *AGTR1* expression was studied in 63 human ovarian cancer specimens. Our results showed that there is a significant positive association between *BRCA1* and *AGTR1* protein expression (Figure 4A and B).

## Discussion

In this study, we report for the first time an association between *BRCA1* and *AGTR1* status in ovarian cancer: (i) *AGTR1* expression was increased in non-*BRCA1*-mutated ovarian cancer, but *BRCA1* dysfunction (such as via *BRCA1* mutation or promoter hypermethylation) was associated with decreased *AGTR1* levels; (ii) there was a positive correlation between *BRCA1* and *AGTR1* expression in ovarian cancer specimens; and (iii) *BRCA1* activation was effective at inducing *AGTR1* expression in primary ovarian cancer cells. These results suggest that *BRCA1* may be a potential trigger for *AGTR1*. Interestingly, the activation effects of *BRCA1* were primarily observed in cells originating from primary ovarian cancer, especially *BRCA1*-mutated ovarian cancer cells, but 293 T and SKOV3 cells were insensitive to the overexpression or knockdown of *BRCA1*. Accordingly, a specific intracellular environment may exist, and *AGTR1* expression is likely to be the long-term result of a complex interaction of multiple factors in *BRCA1*-related ovarian cancer. Notably, a body of evidence suggests that there is extensive crosstalk among *BRCA1* signaling pathways and hormone receptors. For example, the insulin-like growth factor 1 receptor (*IGF1R*) gene is a downstream target for *BRCA1*, as wild-type *BRCA1* expression suppresses promoter activity and endogenous *IGF1R* levels [3]; *BRCA1* can lead to degradation of the progesterone receptor by counteracting the action of progesterone; multiple mechanisms are involved in *BRCA1*-mediated estrogen receptor repression [19,20]. However, to date, there have been few reports about the interactions between *BRCA1* and *AGTR1*. Moreover, mounting evidence indicates that *BRCA1* tumor suppressor gene dysfunction has an important role in promoting cell proliferation and survival [14,21-23]. The mechanism may involve: 1) inducing insulin-like growth factor 1 expression [3,24] in an estrogen receptor α-dependent manner [24,25]; and 2) stimulating progesterone receptor activity by facilitating progesterone binding to the progesterone response elements [26]. *AGTR1* also plays a key role in regulating cell growth and proliferation during the initiation and progression of cancer [27,28]. Therefore, the discovery of *BRCA1*-mediated *AGTR1* expression will stimulate new interest in the study of *BRCA1*-related cellular proliferation, although the details remain unclear. To date, it is not fully understood how *BRCA1* activates *AGTR1* gene expression at the molecular level. However, some insight may be gained by further study, and preliminary data suggest that a direct interaction between miR-155 and *AGTR1* (Additional file 5: Figure S3, methods shown in Additional file 3). Specifically, miR-155 is a regulatory target for *BRCA1*[29,30]; *BRCA1* knockdown results in a two- to three-fold increase in miR-155 levels [29,31], which may be involved in *AGTR1* transcriptional repression, but there are still some details that need to be considered.

## Conclusion

Our results indicate that *BRCA1* may be a potential regulator of *AGTR1* in ovarian cancer cells. Based on these findings, there are some interesting issues that need to be considered in future studies, such as how *BRCA1* affects *AGTR1* transcription and whether other factors could cooperate with *BRCA1* in controlling *AGTR1* expression. Also, the complex interactions between *BRCA1* and *AGTR1* signaling pathways need to be clarified. All of this may improve our understanding of the basic molecular mechanism of *BRCA1*-related ovarian cancer.

## Abbreviations

AGTR1: Angiotensin II type 1 receptor; PCR: Polymerase chain reaction; shRNAs: Short hairpin RNAs

## Competing interests

The authors declare that they have no competing interests.

## Authors’ contributions

DL and QY conceived of the study, participated in its design and drafted the manuscript. DL, FFB and CC carried out data acquisition and interpretation. FFB, CC and CYL participated in the design of the study and performed the statistical analysis. All authors read and approved the final manuscript.

## Supplementary Material

Additional file 1: Table S1 Clinical characteristics for the 15 BRCA1-mutated serous ovarian cancer patients. Click here for file

Additional file 2: Figure S1 Cell proliferation rate of primary ovarian cancer cells. Click here for file

Additional file 3 Supplementary methods. Click here for file

Additional file 4: Figure S2 The efficiency of BRCA1 knockdown and overexpression. Click here for file

Additional file 5: Figure S3 The interaction between miR-155 and AGTR1. Click here for file
