# Supplementary material for: Regulation of angiotensin II type 1 receptor expression in ovarian cancer: a potential role for BRCA1
Source: J Ovarian Res. 2013 Dec 9;6:89. doi: 10.1186/1757-2215-6-89 (PMC4029559; doi:10.1186/1757-2215-6-89)
Supplement: Additional file 1: Table S1 — Clinical characteristics for the 15 BRCA1-mutated serous ovarian cancer patients. [file 1757-2215-6-89-S1.pdf]

Table 1 Clinical characteristics for the 15 BRCA1-mutated serous ovarian cancer patients

Case	Age	Stage[a]	Grade[b]	Gene	Exon	Mutation	AA change	Mutation type[c]
1	70	IIIC	PD	BRCA1	11	c.2331 T > A	p.Y777X	NS
2	47	IIIC	MD	BRCA1	11	c.2311 T > G	p.L771V	MS
3	54	IIIC	PD	BRCA1	11	c.2566 T > C	p.Y856H	MS
4	68	IIIC	MD	BRCA1	11	c.2612 C > T	p.P871L	MS
5	64	IIIC	MD	BRCA1	11	c.2612 C > T	p.P871L	MS
6	57	IA	PD	BRCA1	11	c.2566 T > C	p.Y856H	MS
7	63	IIIC	PD	BRCA1	11	c.2612 C > T	p.P871L	MS
8	56	IIIC	PD	BRCA1	11	c.2709 T > A	p.C903X	NS
9	49	IIB	PD	BRCA1	11	c.3710 C > T	p.A1237V	MS
10	52	IIB	MD	BRCA1	11	c.3109 C > T	p.Q1037X	NS
11	58	IIIC	PD	BRCA1	11	c.2212 G＞A	p.V738I	MS
12	61	IIIC	PD	BRCA1	11	c.2312 T＞C	p.L771S	MS
13	53	IIIC	PD	BRCA1	11	c.2363 T＞C	p.V788A	MS
14	57	IIIC	PD	BRCA1	11	c.2429 A＞G	p.N810S	MS
15	66	IIIC	MD	BRCA1	11	c.2741 A＞G	p.E914G	MS

a: The tumor stages were assessed according to the International Federation of Gynecology and Obstetrics.

b: PD: poorly differentiated; MD: moderately differentiated.

c: NS: nonsense mutation; MS: missense mutation.
