# Supplementary figures and images for: Regulation of angiotensin II type 1 receptor expression in ovarian cancer: a potential role for BRCA1
Source: J Ovarian Res. 2013 Dec 9;6:89. doi: 10.1186/1757-2215-6-89 (PMC4029559; doi:10.1186/1757-2215-6-89)

Supplementary Figure 1

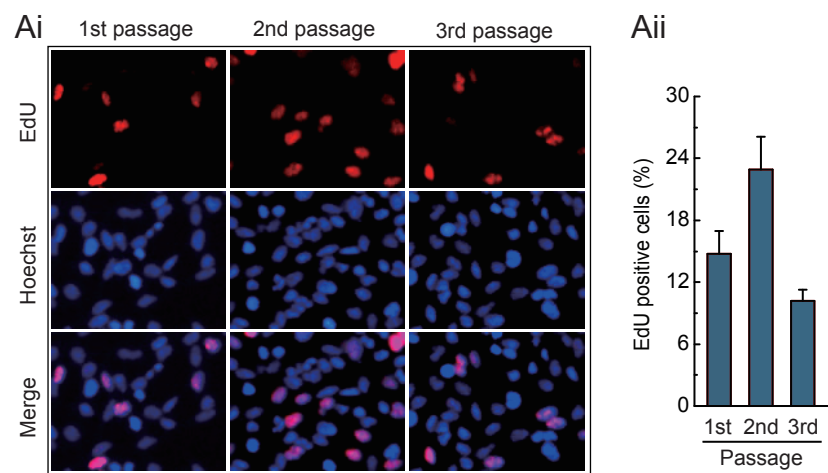

Supplement: Additional file 2: Figure S1 — Cell proliferation rate of primary ovarian cancer cells. [file 1757-2215-6-89-S2.pdf]

Supplementary Figure 2

Ai

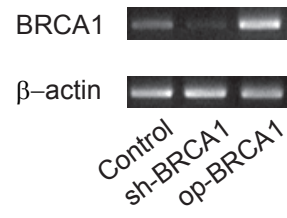

Aii

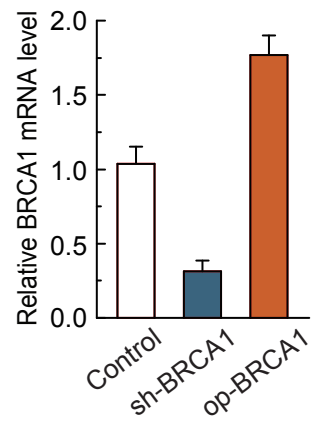

Bi

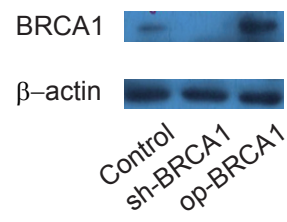

Bii

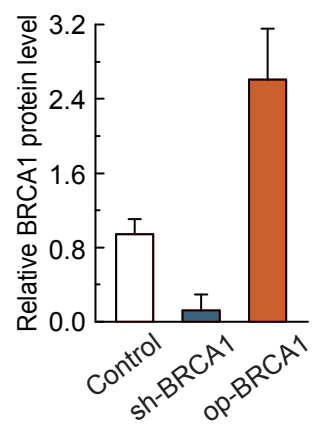

Supplement: Additional file 4: Figure S2 — The efficiency of BRCA1 knockdown and overexpression. [file 1757-2215-6-89-S4.pdf]

Supplementary Figure 3

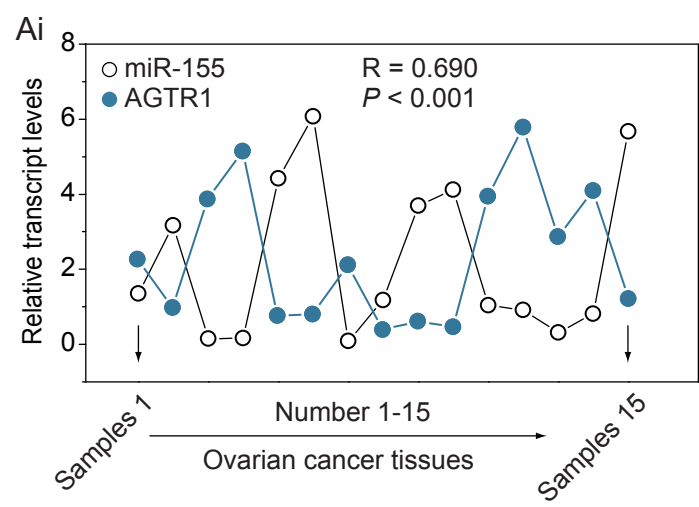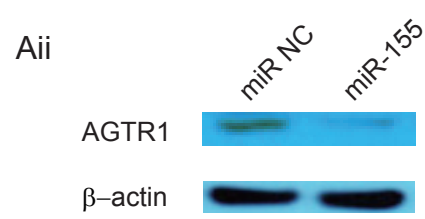

Supplement: Additional file 5: Figure S3 — The interaction between miR-155 and AGTR1. [file 1757-2215-6-89-S5.pdf]
