# Supplementary material for: Regulation of angiotensin II type 1 receptor expression in ovarian cancer: a potential role for BRCA1
Source: J Ovarian Res. 2013 Dec 9;6:89. doi: 10.1186/1757-2215-6-89 (PMC4029559; doi:10.1186/1757-2215-6-89)
Supplement: Additional file 3 — Supplementary methods. [file 1757-2215-6-89-S3.pdf]

Supplementary Methods

Cell proliferation assay

Primary ovarian cancer cells (1st, 2nd and 3rd passage) proliferation was determined using the Cell-Light™ EdU Apollo®643 In Vitro Imaging Kit (Ribobio, Guangzhou, China) following the manufacturer's instructions. Blue, Hoechst 33342 labeling of cell nuclei; red, EdU labeling of nuclei of proliferative cells. Proliferation rate (EdU incorporation rate) was expressed as the ratio of EdU-positive cells to total Hoechst 33342-positive cells.

Semi-quantitative PCR

BRCA1-knockdown efficiency was measured by semi-quantitative PCR. Detailed isolation and reverse-transcribed protocols were established as described in the text (Methods). The specific primer sequences were purchased from Santa Cruz Biotechnology (CA, USA): sc-29219-PR. PCR amplification was performed in a Techne TC-512 gradient thermal cycler (Progene, Techne Ltd., Cambridge, UK). PCR reaction conditions were as follows: 95 °C for 10 min; 32 cycles of 95 °C for 30 s, 60 °C for 30 s and 72 °C for 45 s; followed by an extension reaction at 72 °C for 10 min. The reaction products were analyzed by agarose gel electrophoresis and visualized by UV light after staining with ethidium bromide. The similar results were obtained in primary ovarian cancer cells, 293T cells and SKOV3 ovarian cancer cells. sh: short hairpin RNAs; op: overexpression.

Western blotting

BRCA1-knockdown efficiency was assayed by western blotting. Western blotting analysis of BRCA1 was performed at 48 h after transfection according to standard

protocols. The protein concentration was determined by the Bio-Rad Protein Assay Kit (Bio-Rad, Hercules, CA, USA). Briefly, 30 μg protein was separated by 8% SDS polyacrylamide gels, and transferred to polyvinyl difluoride membranes (Millipore, MA, USA). The membranes were blocked in TBS containing 0.1% Tween-20 and 5% non-fat dry milk for 60 min at room temperature, and incubated with antibody to BRCA1 (sc-642) (1:500; Santa Cruz Biotechnology, CA, USA) overnight at 4 °C. Then, the membranes were washed by PBS-Tween followed by 1 h incubation at room temperature with horseradish peroxidase-conjugated secondary antibody (1:5000; Santa Cruz Biotechnology, CA, USA) and detected using the enhanced chemiluminescence (Amersham Life Science, NJ, USA). The similar results were obtained in primary ovarian cancer cells, 293T cells and SKOV3 ovarian cancer cells. sh: short hairpin RNAs; op: overexpression.

miRNA Transfections

miR mimics (miR-155), and their matched negative control (miR NC) were purchased from Ribobio (Guangzhou, China). The second passage of ovarian cancer cells were plated without antibiotics approximately 24 h before transfections, and miRNA mimics/miR NC transfections were for 48 hours using Lipofectamine™ 2000 (Invitrogen, CA, USA) according to the manufacturer's protocol.

Determination of miR-155

Total RNAs including miRNAs were extracted by using GeneJET RNA Purification Kit (Fermentas) from cultured cells or fresh ovarian cancer tissues following the manufacturer's instructions. Expression of mature miR-155 was determined by Bulge-Loop™ miRNA qRT-PCR Primer Set (Ribobio, Guangzhou, China) with

SYBR Green quantitive real time-PCR (qRT-PCR), U6 snRNA was used as an internal control.
